# Re: investigating the impact of financial concerns on symptoms of depression in UK healthcare workers: data from the UK REACH nationwide cohort study

**DOI:** 10.1192/bjo.2024.30

**Published:** 2024-04-11

**Authors:** Bethany Croak, Danielle Lamb, Sharon A. M. Stevelink

**Affiliations:** Department of Psychological Medicine, King's College London, UK; Department of Applied Health Research, University College London, UK

**Keywords:** Mental health, healthcare workers, financial concerns, depressive disorders, National Health Service

## Abstract

This editorial comments on the paper by Martin McBride and the UK REACH team (published in 2023) investigating financial concerns in UK healthcare workers and depressive symptoms. The research concludes that reporting future financial concerns at baseline increased the odds of depressive symptoms at follow-up around 18 months later. We discuss these findings in the context of the cost-of-living crisis and pay disputes within the NHS, important policy implications and directions for future research.

We read with interest the paper by McBride et al (2023) that introduces novel findings about the relationship between financial concerns and mental health, in particular symptoms of depression, in UK healthcare workers (HCWs).^1^ This is a highly topical subject given the ongoing pay disputes by some of the NHS workforce and the cost-of-living crisis at the time of writing (summer 2023). They conclude that reporting future financial concerns at baseline increased the odds of depressive symptoms at follow-up around 18 months later.

As one of the first pieces of work to examine the relationship between future financial concerns and depression among HCWs, it offers some critical insights and has important policy implications. In particular, despite the recent pay increases of 5–6% for public sector workers,^2^ these do not match current inflation levels (meaning a real-term pay cut) and do not address historic below-inflation public sector pay offers over the past 15 years. Indeed, the ongoing strike action by junior doctors of July 2023 and the concerning numbers of unfilled posts across the sector indicate deep dissatisfaction within the NHS workforce. As the authors rightly remark, the intention to leave and the current problem with staff retention^3^ in the NHS should be incentives to relieve some financial burden.

There are some additional details which would have strengthened this research further and provided even greater insights. Firstly, the occupational groups used in this study differ from those used in the NHS workforce statistics,^3^ which distinguishes between ‘professionally qualified clinical staff’ and ‘support to clinical staff’, a relevant distinction here given that those in the ‘support to clinical staff’ group are often in lower pay bands. (NHS staff are paid using the Agenda for Change pay scale: https://www.healthcareers.nhs.uk/working-health/working-nhs/nhs-pay-and-benefits/agenda-change-pay-rates. Within this, there are bands ranging from 2 to 9. Each band has an entry set point (minimum salary for that band) and a top step point (maximum salary for that band). An NHS staff member's pay band is often dictated by occupation - for example, all newly qualified nurses will start on Band 5.)

Conversely, the authors use staff groups which combine a range of pay bands, a limitation the authors note themselves. For instance, the ‘nursing’ group includes healthcare assistants and nursing associates in bands 3 and 4 who are lower paid than nurses, who start at band 5.^4^ Similarly, ‘admin/estates/other’ could include staff on various pay levels, as admin could comprise staff in band 2, and estates could include estate managers who are in band 7.^4^ Although we understand the necessity to combine occupational groups due to small participant numbers, we suggest it would have been more pertinent to use pay band (rather than occupation), given that this paper focuses on financial concerns. In general, working adults in the UK with the lowest income have the highest rates of moderate to severe depressive symptoms,^5^ and more specifically, lower-paid HCWs are also at higher risk of poorer outcomes.^6^

Secondly, the relationship between employment, income and mental health is complex and multifaceted, which has not been fully explored in this paper – an important consideration given that financial concerns are inherently linked to income. Evidence from the Adult Psychiatric Morbidity Survey (2014) suggests the median income of individuals with anxiety and depression was two-thirds (68%) of those without these mental disorders.^7^ Whereas the authors did control for baseline depression symptoms, future research could include more in-depth analysis to understand the interplay between mental health and low income, especially as no ‘pay band’ variable was used on this occasion. In addition, no other mental health predictors were accounted for in the models used such as post-traumatic stress disorder (PTSD). As serious mental illness can lead to reduced functioning, including the inability to work, it would be pertinent to explore this in future research. Furthermore, it would be useful to know contract type, such as those who work only bank/locum/agency shifts or are on temporary contracts. These are all types of precarious employment which we know are associated with adverse mental health outcomes.^8^

We understand that the authors wished to examine the relationship between future financial concerns at baseline (December 2020–March 2021) and depression symptoms at follow-up (June 2022–October 2022). At baseline, participants were asked ‘How worried are you about your future financial situation?’ not their current situation. We suggest that to assess the impact of future financial concerns more accurately, current financial concerns at baseline should be measured and adjusted for, as the authors did for baseline depression symptoms. This is particularly important given that during the baseline period (December 2020–March 2021), the Coronavirus Job Retention Scheme (known as furlough) was still in place, and although it provided income, individuals may have been concerned about their financial situation, especially in industries such as hospitality that suffered during the pandemic. This may have had an impact on healthcare workers’ concern about their household income if a household member was furloughed or had other pandemic-related job concerns. As household income was not asked for in the UK REACH surveys, it was not possible for the UK REACH team to control for this, but it should be a consideration to include in future waves of UK REACH data collection. Relatedly, it would be relevant to include variables such as relationship status or caring responsibilities, both of which could impact financial concerns as well as mental health. In fact, previous research shows that during COVID-19, working parents had higher levels of financial insecurity compared with those without children.^9^

This paper by Martin McBride and the UK REACH team^1^ has the potential to inform NHS policy and, as discussed, this might include policies to relieve the financial burden on NHS staff. Although pay is important, feedback from staff indicates that getting the basics right to improve working conditions is also important to NHS staff, such as having access to hot food on shift and adequate breaks,^10,11^ as summarised by NHS CHECK and the Policy Institute at King's College London.^12^ Indeed, free parking and affordable food on site are practical matters that would reduce this burden. Therefore, this work should be viewed as one important piece of the puzzle and, taken as a whole, research into NHS staff wellbeing can have positive outcomes for both NHS staff and also for patient care. Finally, we thank the UK REACH team for this timely and interesting research.
